# ASO Author Reflections: Lymphoedema Related to Inguinal and Ilioinguinal Lymphadenectomy for Melanoma

**DOI:** 10.1245/s10434-024-15216-w

**Published:** 2024-03-27

**Authors:** T. S. Lee, A. J. Spillane

**Affiliations:** 1 Melanoma Institute Australia, Sydney, Australia; 2 Royal North Shore Hospital, Sydney, Australia; 3 University of Sydney, Sydney, Australia; 4 Mater Misericordiae Hospital, North Sydney, Australia

## Past

Lymphoedema is a feared side effect of lymphadenectomy, often resulting in chronic discomfort, infection, and reduced function in the affected individual.^1^ Conflicting reports of the prevalence and severity of leg lymphoedema following inguinal (IL) and ilioinguinal lymphadenectomy (IIL) for metastatic melanoma^2^ increases uncertainty for surgeons, who wish to maximise disease control and minimise the potential of extra morbidity related to pelvic dissection.

## Present

The Evaluation of Groin Lymphadenectomy Extent for Melanoma (EAGLE FM) study aimed to determine whether there was a difference in disease-free survival at 5 years after randomisation to IL or IIL when preoperative positron emission tomography/computed tomography (PET/CT) showed no distant or pelvic disease. The study included secondary endpoints of lymphoedema prevalence and severity. The study closed early and did not accrue fully. Despite a trend for patients who had IIL to have greater lymphoedema prevalence than IL at all follow-up timepoints up to 24 months, our study did not have the statistical evidence to support an overall difference between the surgical groups.^3^ Lymphoedema severity was mostly mild.

## Future

EAGLE FM remains the largest, prospective, randomised controlled trial addressing the extent of surgery for inguinal nodal metastatic melanoma, and follow-up for the primary and other survival outcomes is still ongoing. Rapid changes in systemic therapy for stage III melanoma and cessation of completion lymph node dissection for positive sentinel node biopsy after the results of MSLT II^4^ and DeCOG SLT^5^ have already resulted in the decline of patients with, or at risk of, lymphoedema. However, access to systemic therapies in the management of melanoma is unequal worldwide, and for many patients, lymphadenectomy is still necessary for disease control. Our results show that in a monitored environment, leg lymphoedema can be detected early, irrespective of the extent of surgery, and managed accordingly to minimise severity.
